# Utility of continuous glucose monitoring during pancreatic surgery in patients with congenital hyperinsulinism

**DOI:** 10.3389/fendo.2026.1788026

**Published:** 2026-03-11

**Authors:** Sarah Worthington, Chris Worth, Sameera Auckburally, Matthew Bowler, Benoit Beauve, Elaine O’Shea, Ross J Craigie, Maria Salomon-Estebanez, Indraneel Banerjee

**Affiliations:** 1 Department of Paediatric Endocrinology, Royal Manchester Children’s Hospital, Manchester, United Kingdom; 2 Department of Paediatric Anaesthesia, Royal Manchester Children’s Hospital, Manchester, United Kingdom; 3 Department of Paediatric Surgery, Royal Manchester Children’s Hospital, Manchester, United Kingdom; 4 Faculty of Biology, Medicine and Health, University of Manchester, Manchester, United Kingdom

**Keywords:** CGM, congenital hyperinsulinism, continuous glucose monitoring, hypoglycaemia, pancreatic surgery, hyperglycaemia

## Abstract

**Introduction:**

Congenital hyperinsulinism [CHI] is a rare disorder characterised by hypoglycaemia secondary to excessive insulin secretion from the pancreas. The aim of management of CHI is to prevent severe and persistent hypoglycaemia which can lead to poor neurodevelopmental outcomes. Pancreatic surgery can play a role in the improvement of glycaemic safety in focal and diffuse CHI. Continuous glucose monitoring [CGM] is increasingly used in diabetes management, yet its application in CHI, particularly during perioperative settings, remains underexplored.

**Objective:**

This study aimed to evaluate the utility and accuracy of CGM during pancreatic surgery in CHI patients.

**Research design and methods:**

A mixed methods observational study was conducted over three years involving 13 patients undergoing either focal lesionectomy or subtotal pancreatectomy. CGM was performed using the Dexcom G6 device, paired with point-of-care capillary blood glucose measurements to assess accuracy. Qualitative feedback from healthcare professionals and quantitative analysis of CGM data were collected.

**Results:**

Hyperglycaemia was observed in 8 patients, influenced by surgical stress and dextrose administration. CGM analysis revealed a median perioperative glucose level of 9.2 mmol/L, with a mean absolute relative difference [MARD] of 19.3% compared to capillary samples. The majority of professionals [94%] reported CGM as helpful in the perioperative period, highlighting its role in guiding prompt adjustment of dextrose infusions and reducing blood sampling frequency.

**Conclusion:**

CGM demonstrates utility in monitoring glycaemic trends and managing hyperglycaemia during pancreatic surgery in CHI patients. The integration into perioperative care may further enhance surgical safety, extending the range of clinical applications of CGM.

## What is already known on this topic

CHI causes profound and recurrent hypoglycaemia, maintaining glycaemic stability during pancreatic surgery can be difficult due to rapidly shifting glucose levels. CGM has been shown to be useful in everyday management of CHI, but its role and reliability in surgery has not been established, creating a clear evidence gap.

## What this study adds

This study shows that CGM can function effectively during pancreatic surgery and providing a continuous trend in glucose levels that complement standard testing. It also reveals that CGM identifies rapid glycaemic changes that standard sampling may miss, which supports more responsive intraoperative management of glucose.

## How this study might affect research, practice or policy

The findings support the use of CGM as an adjunct to routine glucose monitoring during CHI surgery to improve intraoperative decision making. It also justifies larger studies to inform future perioperative guidelines for managing rare hypoglycaemia diseases.

## Introduction

Congenital hyperinsulinism (CHI) is a rare disease of childhood, characterised by hypoglycaemia secondary to excessive and dysregulated insulin secretion. The incidence of CHI varies by country but is at least 1:28000 in the United Kingdom (1). The severe and recurrent hypoglycaemia experienced by these patients can result in poor neurodevelopmental outcomes. Indeed, it has been reported that 15-48% of patients with CHI have had abnormal neurodevelopment (2, 3); the figure being highest in those where there has been a delay in diagnosis and treatment.

Patients with a genetic cause for CHI can be categorised into two main groups: diffuse and focal (4). Focal CHI occurs following the inheritance of a recessively acting mutation on the paternal allele of *ABCC8/KCNJ11* with loss of maternal heterozygosity and is defined by the finding of a localised collection of abnormal tissue within the pancreas (5). The finding of genetic results suggestive of focal CHI prompts ^18^FluoroDOPA PET CT scanning and, if a focal lesion is confirmed, focal lesionectomy surgery is frequently undertaken with a good chance of cure (6). Contrastingly, diffuse CHI can be defined by its specific histological features but practically is diagnosed in the absence of focal hyperinsulinism and is characterised by abnormalities within the β-cells across the entire pancreas (6). Management in diffuse CHI is primarily medical but subtotal pancreatectomy may be required if glycaemic safety cannot be achieved with medication alone. Management following 95% subtotal pancreatectomy is unpredictable and individual to each patient; ranging from ongoing hypoglycaemia to insulin-dependent diabetes (7).

Monitoring of blood glucose is the cornerstone of CHI management: this has historically been performed via intermittent fingerprick glucose testing in both the inpatient and outpatient setting. However, intermittent sampling caries a risk of missed hypoglycaemia between tests and offers no information on glycaemic trends. Alternatively, continuous glucose monitoring [CGM] is performed by minimally invasive devices inserted subcutaneously to monitor interstitial glucose levels and transmit this data to a nearby receiver device at frequent intervals. CGM is now widely used in patients living with diabetes and has good efficacy in the reduction of hypoglycaemia (8). CGM devices are increasingly being used for patients with CHI (9, 10). Although accuracy is suboptimal (11, 12) and efficacy to reduce hypoglycaemia is not yet proven (13), patient feedback of CGM in CHI in the outpatient setting is mixed but largely positive (14). However, despite increasingly widespread use, and recent publications demonstrating the feasibility of CGM use perioperatively for those with diabetes mellitus (15), there have been no descriptions of the use of CGM in the perioperative or inpatient setting for patients with CHI.

Our team, working in the highly specialised Northern Congenital Hyperinsulinism service (NORCHI) at Royal Manchester Children’s Hospital, has extensive experience in using CGM for patients with CHI undergoing pancreatic surgery. We undertook a mixed methods study to determine the perceived value and accuracy of CGM in the perioperative period for patients with CHI, with the aim of improving the care of those undergoing pancreatic surgery and to provide information to the international community.

### Aims

This study aimed to observe, understand and describe the utility of CGM in pancreatic surgery for patients with CHI. Primary aims were:

1. To describe and analyse the accuracy of CGM during pancreatic surgery.
2. To understand healthcare professionals’ opinions of the utility of CGM in pancreatic surgery.

## Methods

A mixed methods observational study with qualitative questionnaires submitted to healthcare professionals was combined with conventional accuracy analyses of perioperative CGM values. Over a three-year period (Jan 2021 to Aug 2023) 13 patients with CHI undergoing either focal lesionectomy or subtotal pancreatectomy received monitoring with a Dexcom G6 CGM device, applied at least 24 hours prior to surgery to ensure maximal accuracy. CGM monitoring was also used during the insertion of 6 central venous catheter procedures and during a laparotomy procedure in the same group of patients. CGM devices continued to be used for the duration of clinical need to monitor glycaemic patterns and replaced at the end of the sensor operational lifespan. Patients achieving cure following focal lesionectomy (focal CHI) discontinued CGM use after demonstration of satisfactory fasting tolerance. Patients undergoing subtotal pancreatectomy (diffuse CHI) continued to use CGM at discharge from the hospital.

For the purposes of the study and for simplicity, the terms hypo and hyperglycaemia on CGM were deemed to be equivalent to the interpretation of such terms with blood glucose measurements.

Written consent was taken from all families, in line with ethical approval from the ethics committee of the University of Manchester and the Health Research Authority of the National Health Service (REC reference 07/H1010/88). Professionals involved in the perioperative care of patients were approached to understand opinions on the utility of CGM in this period. Each professional was asked four questions (Likert scale 1-5): three questions required a rating of the clinical utility of the CGM in various fields and one open question allowed for discussion of opinions on the utility of CGM in the perioperative period (**Appendix 1**). The perioperative period was defined as starting when the patient entered the anaesthetic room and ended one hour after leaving postoperative recovery.

Glucose levels from the CGM devices were paired with point-of-care capillary samples taken in the anaesthetic room, operating theatre and postoperative recovery/ward in a pairing process described in depth elsewhere (11). Point of care testing (POCT) capillary samples were analysed using a Nova Statstrip™ Point of Care testing device or Werfen Gem 5000™ blood gas analyser. The intention of the timing of capillary and/or blood glucose sampling was based on multiprofessional perception of clinical need. Paired values were analysed for standard accuracy measures and plotted on the Hypoglycaemia Error Grid (HEG) using Python 3.1.1. This provides information on the specific accuracy of CGM for patients with CHI undergoing pancreatic surgery as well as a clinical context to the accuracy values. Hypoglycaemia was defined as <3.5mmol/L as per standard UK practice (17) and hyperglycaemia as >10mmol/L. A rapid increment in CGM glucose identified by visual inspection was defined as a glucose increment of 2.5 mmol/L from the time of induction of anaesthesia to the time of removal of a focal lesion or excision of diffuse pancreas.

## Results

**Figure 1:**
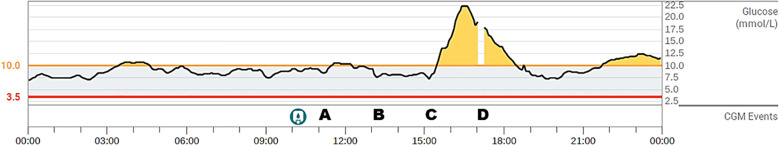
CGM trace of a patient undergoing focal lesionectomy. **(A)** Anaesthetic induction **(B)** Surgery started, **(C)** Focal lesion removed, **(D)** Surgery completed.

**Figure 2:**
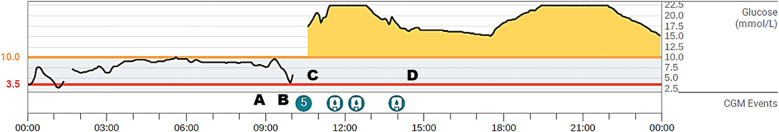
CGM trace of a patient undergoing a 95% subtotal pancreatectomy. **(A)** Anaesthetic induction **(B)** Surgery started, **(C)** 95% Subtotal Pancreatectomy, **(D)** Surgery completed.

**Figure 3:**
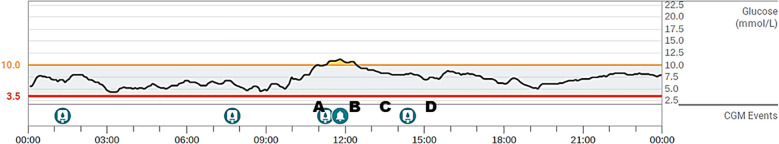
CGM trace of a patient undergoing focal lesionectomy. **(A)** Anaesthetic induction **(B)** Surgery started, **(C)** Focal lesion removed, **(D)** Surgery completed.

**Figure 4:**
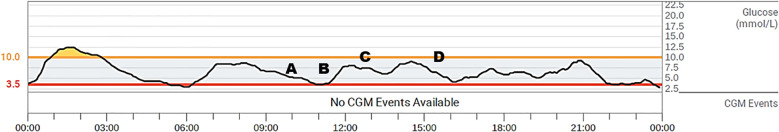
CGM trace of a patient undergoing a 95% subtotal pancreatectomy. **(A)** Anaesthetic induction **(B)** Surgery started, **(C)** 95% Subtotal Pancreatectomy, **(D)** Surgery completed.

During the study period described, CGM was used by the research team in the perioperative period to support the medical and surgical management of 13 patients (age range 4 weeks to 22 months) undergoing pancreatic surgery (focal lesionectomy = 11, subtotal pancreatectomy = 2) (**Table 1**). In all patients’ peri-operative CGM outputs were recorded (**Figures 1-4**) on the basis of specific anaesthetic and surgical events.

**Table 1:** Patient Demographics with age at procedure, type of pancreatic surgery and pre-operative medications.

Patient	Age at procedure	Sex	Genetic mutation	Surgery	Pre-operative medications
1	4 months	Male	*Paternally inherited* heterozygous *ABCC8*	Focal Lesionectomy	15% Glucose infusion, Octreotide
2	8 weeks	Male	*Paternally inherited* heterozygous *ABCC8*	Focal Lesionectomy	50% Glucose infusion, Octreotide, Glucagon
3	3 months	Male	*Paternally inherited* heterozygous *ABCC8*	Focal Lesionectomy	50% Glucose infusion, Octreotide, Glucagon
4	5 months	Male	Beckwith Wiedemann syndrome	Focal Lesionectomy	50% Glucose infusion, Octreotide
5	6 weeks	Female	*Homozygous recessive ABCC8*	Subtotal Pancreatectomy	20% Glucose infusion, Octreotide, Glucagon
6	8 weeks	Female	*Paternally inherited* heterozygous *KCNJ11*	Focal Lesionectomy	20% Glucose infusion, Octreotide
7	13 months	Female	*Paternally inherited* heterozygous *ABCC8*	Focal Lesionectomy	20% Glucose infusion, Octreotide, Glucagon
8	7 weeks	Male	*Paternally inherited* heterozygous *ABCC8*	Focal Lesionectomy	50% Glucose infusion, Octreotide, Glucagon
9	22 months	Male	*Paternally inherited* heterozygous *ABCC8*	Focal Lesionectomy	10% Glucose infusion, Octreotide, Glucagon
10	10 weeks	Female	*Paternally inherited* heterozygous *ABCC8*	Focal Lesionectomy	20% Glucose infusion, Octreotide, Glucagon
11	4 weeks	Female	*Homozygous recessive ABCC8*	Subtotal Pancreatectomy	20% Glucose infusion, Octreotide Glucagon
12	7 weeks	Male	*Paternally inherited* heterozygous *ABCC8*	Focal Lesionectomy	30% Glucose infusion, Octreotide, Glucagon
13	15 weeks	Female	*Paternally inherited* heterozygous *ABCC8*	Focal Lesionectomy	20% Glucose infusion, Octreotide Glucagon

Genetic mutations are grouped into categories suggestive of focal and diffuse CHI. Patients with paternal heterozygous mutations underwent 18-fluoro-dopa PET-CT scanning to identify focal lesion prior to focal lesionectomy. All patients received octreotide prior to surgery; octreotide was weaned off in anticipation of surgery with glucose stabilisation achieved by a combination of intravenous dextrose and glucagon.

### CGM detection of hyperglycaemia

From the 13 patients included in this study, 8 experienced varying degrees of hyperglycaemia during the perioperative period (**Appendix 2**). Hyperglycaemia was identified in three settings during pancreatic surgery: hyperglycaemia secondary to high dextrose provision; surgical stress (temporally associated with anaesthetic and/or surgery); and resection of focal lesion and/or subtotal pancreatectomy (observed as a rapid increment in glucose temporally associated with resection of lesion/diffuse pancreatic tissue (**Figures 1**, **2**).

The timing of hyperglycaemia was relevant to the surgical procedure in two distinct ways. In 2 (15%) patients prolonged hyperglycaemia secondary to surgical stress was identified early; this enabled a reduction in amount of dextrose in intravenous fluid (**Figures 1**, **3**).

In 4 (30%) patients, CGM in the perioperative period demonstrated a hyperglycaemic rapid increment following subtotal pancreatectomy or successful focal lesionectomy (**Figures 1**, **2**). In surgery for focal disease, combined with real-time frozen section histopathology, this glycaemic shift added information to confirm successful focal lesionectomy. Similarly, in subtotal pancreatectomy, post-resection CGM rapid increments led to further rapid down titration of dextrose.

In **Figure 1**, a rapid glucose increment correlated with complete resection of a focal lesion with no remnant abnormal tissue. Likewise, in **Figure 2** hyperglycaemia was noted following subtotal pancreatectomy. By contrast, in patients in **Figures 3**, **4**, a clear glycaemic rapid increment was not observed. In **Figure 3**, anaesthetic induction coincided with rising glucose levels, indicating surgical stress. In **Figure 4** in a patient with diffuse CHI undergoing subtotal pancreatectomy, hyperglycaemia was tightly regulated by dextrose titration with no appreciable rapid increment and a tendency to hypoglycaemia.

The pattern of hyperglycaemia was also observed in five patients with CHI undergoing central venous catheterisation and one patient having laparotomy; here a glucose rapid increment was synchronous with induction of anaesthesia in three patients, coinciding with surgical/anaesthetic stress.

### Glucose trends and accuracy of CGM

**Figure 5:**
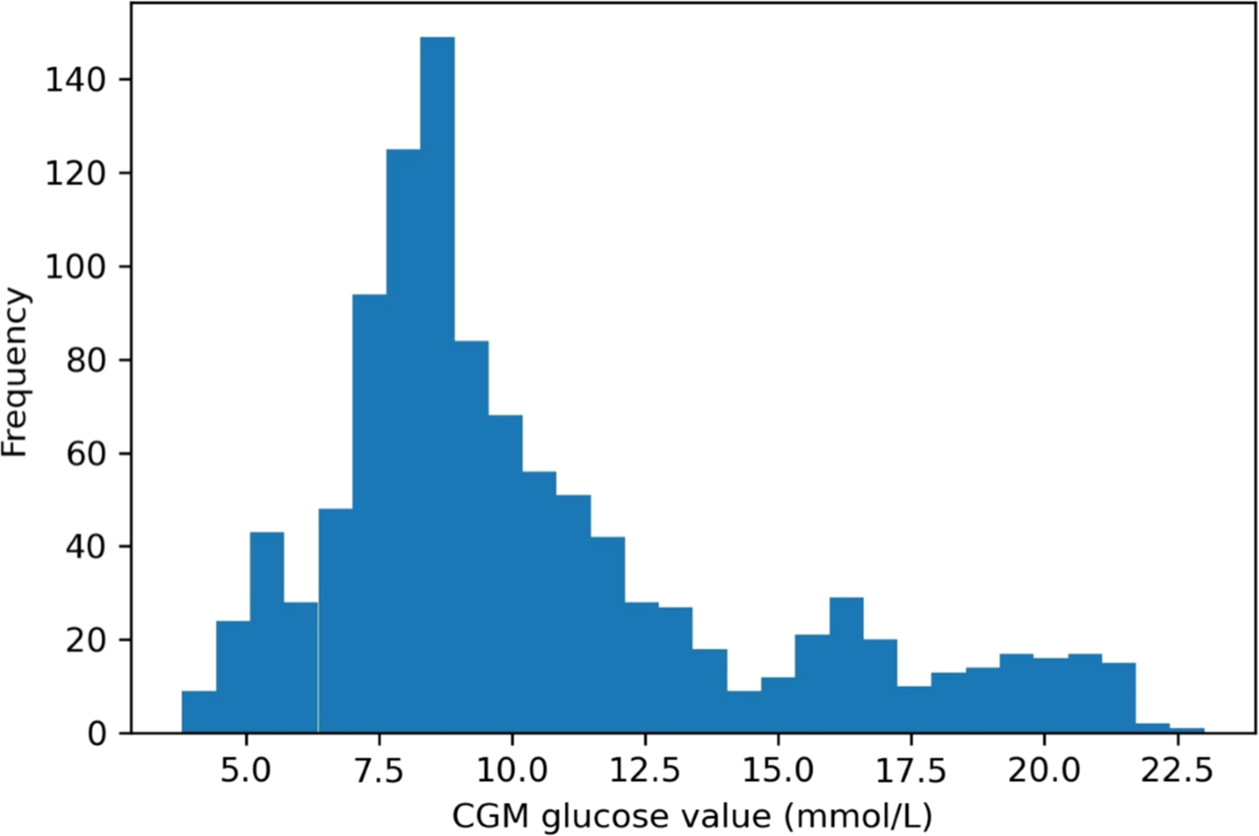
Distribution of CGM glucose values in the perioperative period. A large proportion of CGM glucose levels (421, (39%)) were in the hyperglycaemic range (> 10 mmol/L) and only a few values were below 7.5 mmol/L.

**Figure 6:**
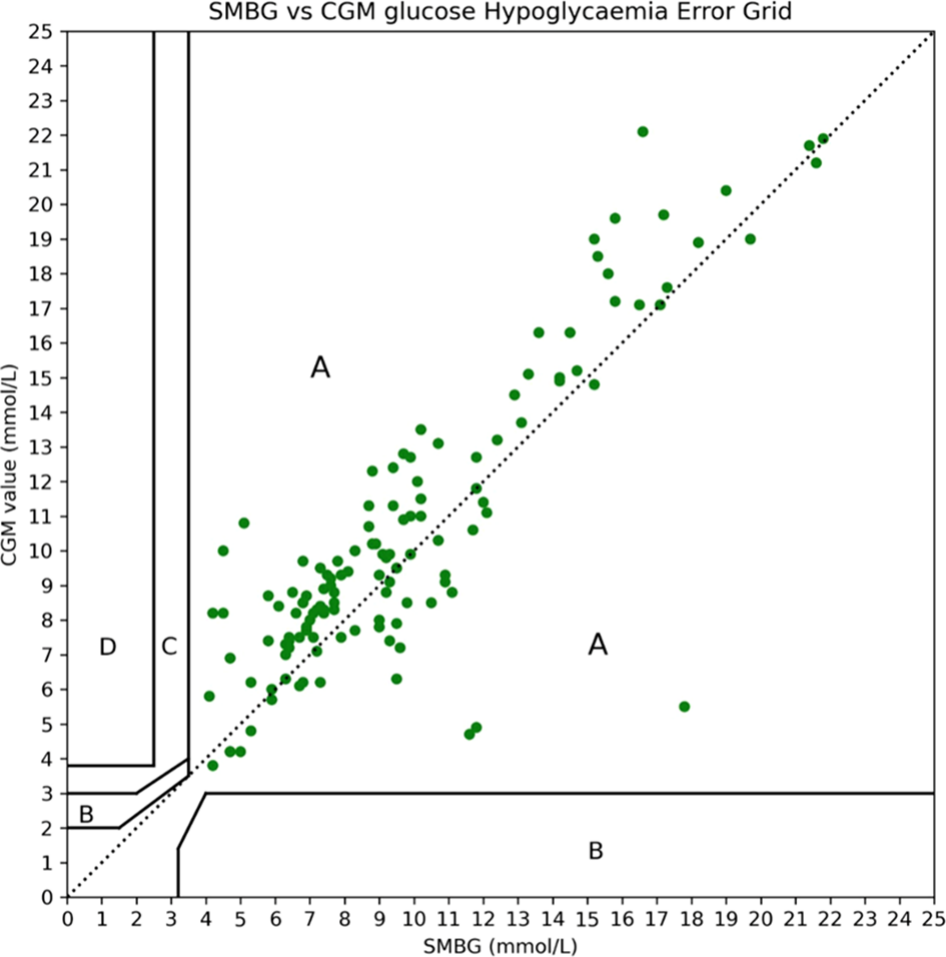
Hypoglycaemia Error Grid (HEG) plotted with perioperative CGM vs SMBG glucose values. All values lie within Zone A and the error is thus classed as no risk.

CGM data analysis in all patients yielded a total of 1091 CGM data points, representing 40 hours of perioperative glycaemic monitoring. with a median (interquartile range) peri-operative glucose 9.2 (4.1) mmol/L with no values in the hypoglycaemic (<3.5mmol/L) range (**Figure 5**). CGM values were matched with 129 POCT blood glucose levels within a predefined 5-minute interval. Mean absolute relative difference (MARD) was 19.3% with a mean absolute difference (MAD) of 1.8mmol/L. The Dexcom G6 tended to overestimate glucose values with a mean difference of +0.5mmol/L. When plotted on the Hypoglycaemia Error Grid (HEG) (11), 100% of values were in Zone A (**Figure 6**) and thus classified as posing no clinical risk.

### Questionnaire review

Of the 21 professionals approached (7 anaesthetists, 2 surgeons, 9 endocrinologists, 3 specialist nurses), 17 professionals completed CGM utility questionnaires. From these 17 (5 anaesthetists, 2 surgeons, 7 endocrinologists, 3 specialist nurses) responses were overwhelmingly positive with all but one respondent reporting CGM as helpful or extremely helpful in the perioperative period (**Table 2**). The single endocrinologist who was neutral on the use of CGM reported concerns about accuracy limiting the utility of CGM for these patients. Generally, responses remained positive (most frequent Likert score 4) over a range of issues relating to CGM in the perioperative period.

**Table 2:** Professional opinion of utility of CGM during the process of pancreatic surgery.

Question 1: How useful was CGM in the intraoperative period on a scale of 1 -5?					
Professional:	Very unhelpful	Not helpful	Neither helpful nor unhelpful	Very helpful	Extremely helpful
Anaesthetists				2	3
Endocrinologists			1	4	2
Surgeons				2	
CHI Specialist Nurses				1	2
Question 2: Did CGM help to guide administration of glucose in the intraoperative/post-operative period? On a scale of 1-5.					
Anaesthetists				2	3
Endocrinologists			1	5	1
Surgeons				2	
CHI Specialist Nurses				1	2
Question 3: Overall, what is your view of clinical utility of CGM in pancreatic surgery?					
Anaesthetists				2	3
Endocrinologists			1	5	1
Surgeons				2	
CHI Specialist Nurses				1	2

Free text provision provided further insights into how different professionals viewed the use of CGM in the paediatric operating room. Anaesthetists found CGM very helpful or extremely helpful, reporting that:

“ It enhances safety for avoiding hyperglycaemia or hypoglycaemia. It prevents the need for continuous sampling of blood from an arterial line. It can be useful for seeing a real time reduction associated with a successful resection”.

Both pancreatic surgeons reported CGM as being very helpful and one commented:

“CGM provides a useful adjunct to histology in terms of identifying whether surgery has been successful”.

All but one of the endocrinologists & specialist nurses felt the CGM was very/extremely helpful, as:

“The ability to identify rapidly changing glucose values in the perioperative period allows for targeted fingerpick checking and pre-emptive strategies to mitigate hypo and hyperglycaemia”, although “accuracy issues” were cited in the neutral comment from the one endocrinologist who disagreed on CGM utility.

Additional comments collectively suggested that CGM did not completely remove the requirement for fingerprick blood glucose sampling but reduced the frequency of sampling and guided timely adjustments in therapies with real-time feedback on changes.

## Discussion

Our observational, mixed methods study of CGM use in patients with CHI undergoing pancreatic surgery demonstrated utility as judged by professionals involved directly with patient care. A tendency to hyperglycaemia, rather than hypoglycaemia, was observed in glycaemic profiles captured by CGM. Accuracy of glucose results correlated with previous study findings (11, 12), suggesting that accuracy was consistent in the surgical setting. As expected, CGM was found to be useful by the majority of professionals involved in surgical patient care.

The use of CGM has been recommended in the intraoperative period in patients with type 1 diabetes (15). However, no previous studies have described CGM utility in the context of pancreatic surgery in patients with CHI. Our results, albeit in a heterogeneous group of CHI patients, provide supportive evidence for extending CGM use beyond home glucose monitoring. While the sample size (n=13) may be construed as small, pancreatic surgery is only undertaken in relatively small proportions of patients. Our study is supplemented by qualitative feedback, enhancing the objective benefits of CGM interpretation. Thus, our study provides a well-defined initial basis from which to design larger CGM studies testing surgical utility and benefit. Nonetheless, given relative small sample size and a single centre bias, our results need careful interpretation and future validation in other studies.

A weakness in our study design is the absence of a uniform fluid and anaesthetic protocol for pancreatic surgery. However, owing to the retrospective nature of the study, the heterogeneity of diagnoses and procedures, and the need for individualisation in a clinical context, we recognised that it would be unrealistic to maintain uniformity through implementation of a rigid protocol. Nonetheless, future studies may wish to concentrate on CGM performance in a protocolised prospective study design, using principles based on our study observations. A further limitation of this study is the heterogeneity of the patient cohort, which includes individuals across a broad age range undergoing different surgical interventions, specifically focal versus subtotal pancreatectomy. This variability introduces potential confounding factors that may influence outcomes and complicate direct comparisons across subgroups. While such diversity was necessary due to the rarity of the condition and the limited number of eligible cases, it nonetheless impacts the generalisability of the findings. Future studies with larger, more homogeneous cohorts or stratified analyses may help to clarify the influence of specific patient or procedural characteristics on clinical outcomes.

It is expected that glucose fluxes observed by CGM are also likely to be observed by frequent capillary blood glucose testing, if high frequency sampling were possible in theatre. However, given the observational nature of our study and relative low number of patients undergoing surgery, it was not possible to assess a comparative group relying solely on capillary blood glucose sampling without the aid of CGM. Future studies comparing cohorts with or without CGM maybe helpful to determine the utility of intraoperative CGM vis-à-vis frequent blood glucose testing.

Episodic hyperglycaemia was a common finding in the perioperative period, correlating with an intraoperative stress response. Here, real-time CGM was useful by providing trend information that enabled the reduction and adjustment of dextrose infusions, thereby preventing large glucose fluxes and the need for continuous insulin infusion as additional therapy. The presence of hyperglycaemia was additionally informative in patients with focal CHI. In some (but not all), successful lesionectomy (with confirmation by intraoperative frozen section biopsy) correlated with a distinct upward shift in glucose. However, given the ability to make real-time anticipatory dextrose reductions throughout surgery, a large increment in glucose levels is not expected in all patients. Therefore, the absence of a large glucose increment following focal lesionectomy is not a rigid indicator of surgical success.

The accuracy of CGM devices in the perioperative setting closely approximated accuracy in the outpatient setting (2, 11) with a tendency towards overreading of glucose levels. Although accuracy of CGM was lower than values reported in people living with diabetes (16), MARD was similar to values obtained in patients with CHI (17), reiterating the unreliability of MARD as a measure of sensor accuracy. It is acknowledged that MARD is not a reliable indicator for CGM accuracy in CHI; several factors such hyperinsulinism, rapid fluctuations and reading lag are speculative causes. It is also recognised that CGM sensors are developed for diabetes, not CHI and their use is undertaken in an unlicensed capacity. While point accuracy remained suboptimal, clinical accuracy, as judged by HEG analysis, was high, as all values, including those in the hyperglycaemic range, were of short duration, clinically acceptable and therefore in the safe range. However, for further confirmation of glycaemic control, concomitant capillary blood glucose testing would be recommended in preparation for potential insulin therapy in the post-pancreatectomy period. Although one endocrinologist retained a neutral view on CGM utility in surgery, citing accuracy concerns, qualitative experiential data derived from professionals involved in pancreatic surgery added confidence in CGM utility. Therefore, barring the minor limitation of a positive bias, CGM utility in the peri-operative period suggests its potential application as a safe and effective tool for monitoring and managing glycaemic trends during pancreatectomy.

## Conclusion

CGM may be useful in demonstrating episodes of hyperglycaemia during subtotal pancreatectomy and focal lesionectomy in patients with CHI. Although point accuracy of CGM in CHI remains suboptimal, intra-operative monitoring appears to receive wide acceptance by professionals involved in the surgical care of CHI patients. CGM has the potential for integration into perioperative care to enhance surgical safety, extending the range of clinical applications of CGM; however, larger datasets in different centres will be required for validation. Future studies examining surgical benefits of CGM may focus on specific pancreatic surgical procedures in patients with CHI.

## Data Availability

The raw data supporting the conclusions of this article will be made available by the authors, without undue reservation.
