# Supplementary material for: Utility of continuous glucose monitoring during pancreatic surgery in patients with congenital hyperinsulinism
Source: Front Endocrinol (Lausanne). 2026 Mar 11;17:1788026. doi: 10.3389/fendo.2026.1788026 (PMC13012931; doi:10.3389/fendo.2026.1788026)

## Appendix 1

Questionnaire completed by professionals: Anaesthetists, Surgeons, Endocrinologists and Congenital Hyperinsulinism specialist nurses.


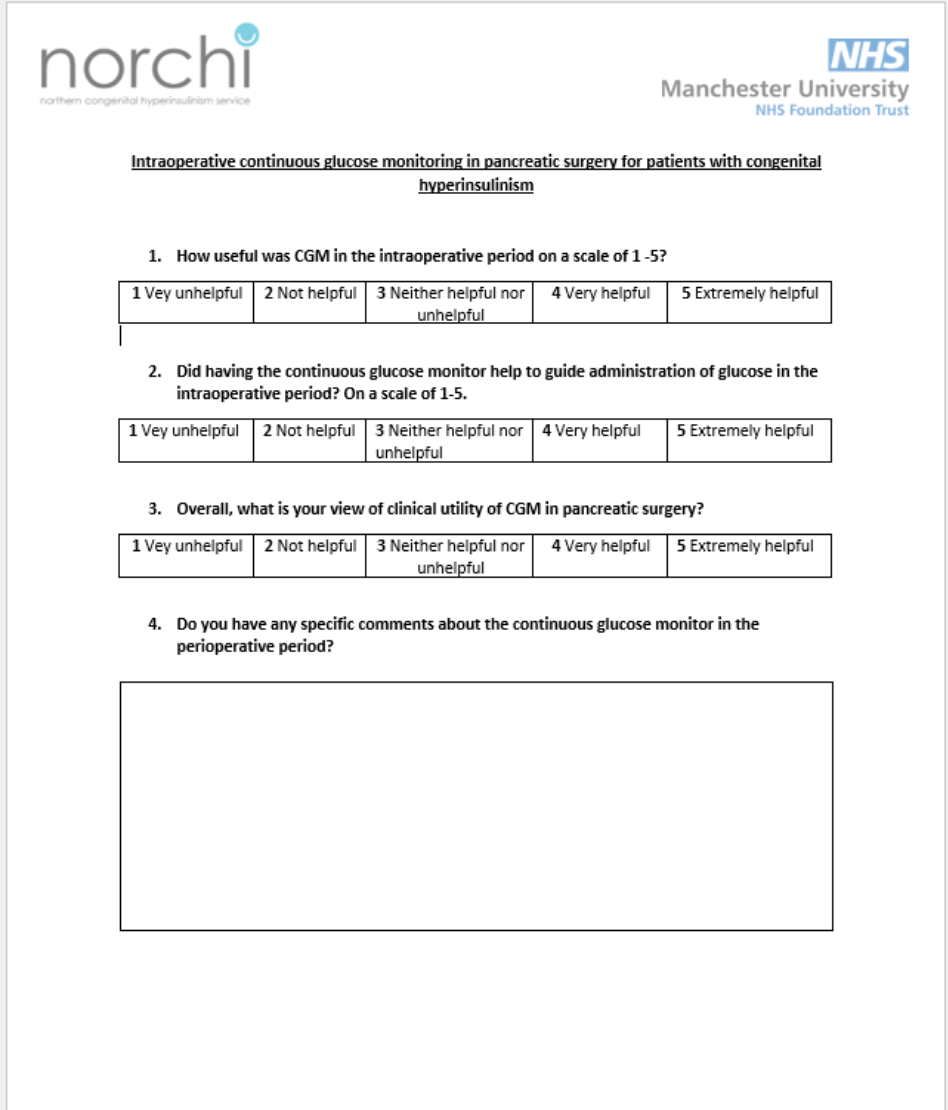

Supplement: Supplementary file 1 [file DataSheet1.docx]
