# Supplementary material for: Utility of continuous glucose monitoring during pancreatic surgery in patients with congenital hyperinsulinism
Source: Front Endocrinol (Lausanne). 2026 Mar 11;17:1788026. doi: 10.3389/fendo.2026.1788026 (PMC13012931; doi:10.3389/fendo.2026.1788026)
Supplement: Supplementary file 2 [file DataSheet2.docx]

## Appendix 2

CGM Data from the day of surgery for participants undergoing a focal lesionectomy, 95% subtotal Pancreatectomy or other surgical intervention (line insertion/exploratory laparotomy).


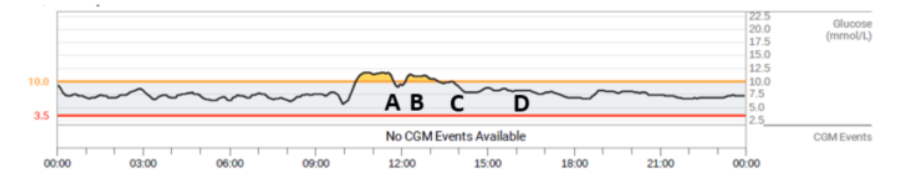


**Patient 1** - CGM trace in patient #1 undergoing Focal Lesionectomy. During surgery, the patient received continuous intravenous dextrose and did not receive medication causing hyperglycaemia. A = Anaesthetic induction B = Surgery started, C= Focal lesion removed, D = Surgery completed.


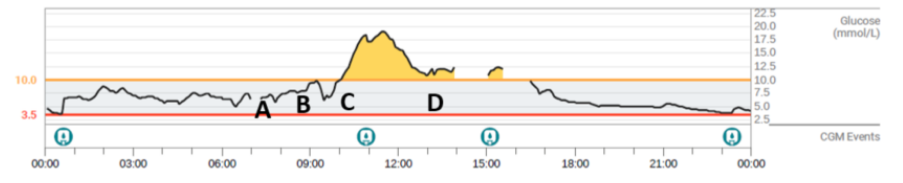
**Patient 2**: CGM trace in patient #2 undergoing Focal Lesionectomy. During surgery, the patient received continuous intravenous dextrose and did not receive medication causing hyperglycaemia. A = Anaesthetic induction B = Surgery started, C= Focal lesion removed, D = Surgery completed.


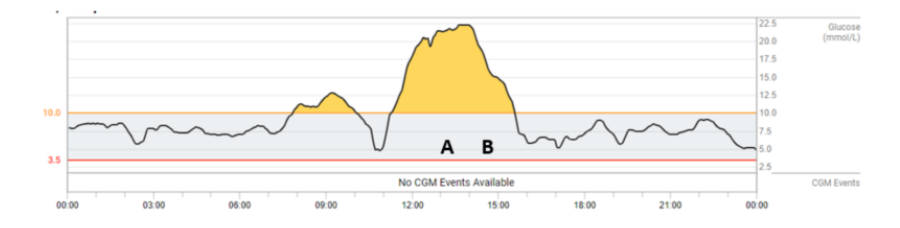
**Patient 2:** CGM trace in patient #2 undergoing Line insertion, labelled as follows: A = Surgery started, B = surgery completed.


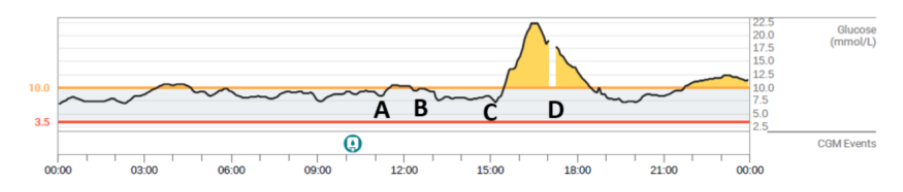
**Patient 3:** CGM trace in patient #3 undergoing Focal Lesionectomy. During surgery, the patient received continuous intravenous dextrose and did not receive medication causing hyperglycaemia. A = Anaesthetic induction B = Surgery started, C= Focal lesion removed, D = Surgery completed.


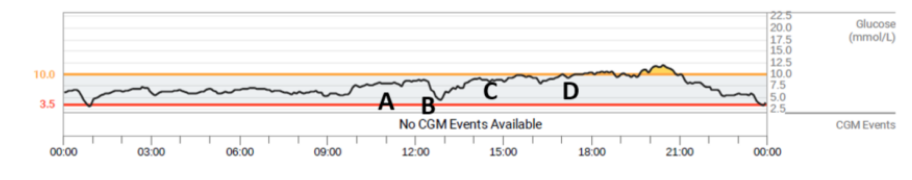
**Patient 4:** CGM trace in patient #4 undergoing Focal Lesionectomy. During surgery, the patient received continuous intravenous dextrose and did not receive medication causing hyperglycaemia. A = Anaesthetic induction B = Surgery started, C= Focal lesion removed, D = Surgery completed.


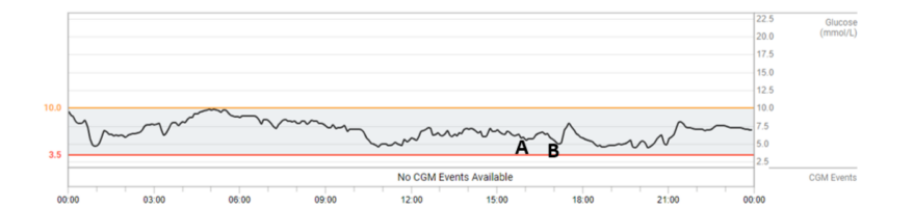
**Patient 4:** CGM trace in patient #4 undergoing Line insertion, labelled as follows: A = Surgery started, B = surgery completed.


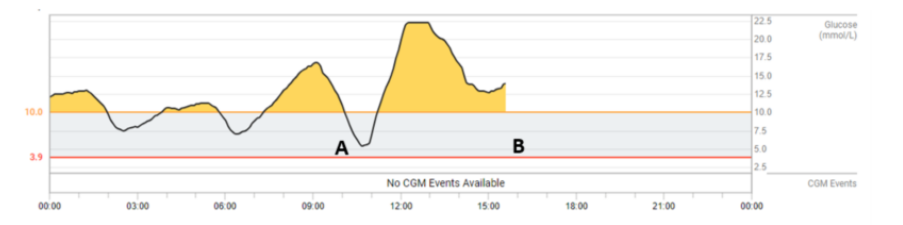
**Patient 4:** CGM trace in patient #4 undergoing Laparotomy, labelled as follows: A = Surgery started, B = surgery completed,


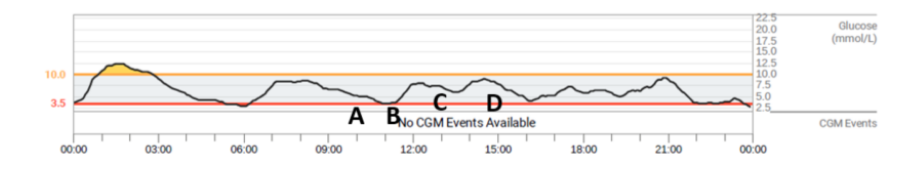
**Patient 5:** CGM trace in patient #5 undergoing a 95% subtotal Pancreatectomy. During surgery, the patient received continuous intravenous dextrose and did not receive medication causing hyperglycaemia. A= Anaesthetic induction B = Surgery started, C= 95% Subtotal Pancreatectomy, D = Surgery completed.


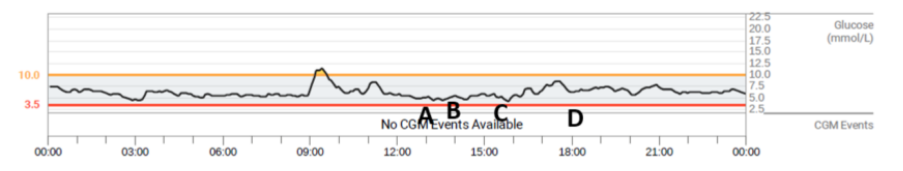


**Patient 6:** CGM trace in patient #6 undergoing Focal Lesionectomy. . During surgery, the patient received continuous intravenous dextrose and did not receive medication causing hyperglycaemia. A = Anaesthetic induction B = Surgery started, C= Focal lesion removed, D = Surgery completed.


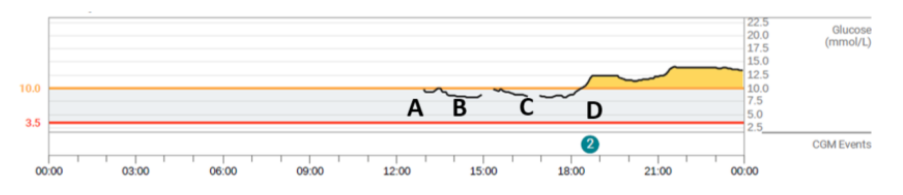


**Patient 7:** CGM trace in patient #7 undergoing Focal Lesionectomy. . During surgery, the patient received continuous intravenous dextrose and did not receive medication causing hyperglycaemia A = Anaesthetic induction B = Surgery started, C= Focal lesion removed, D = Surgery completed.


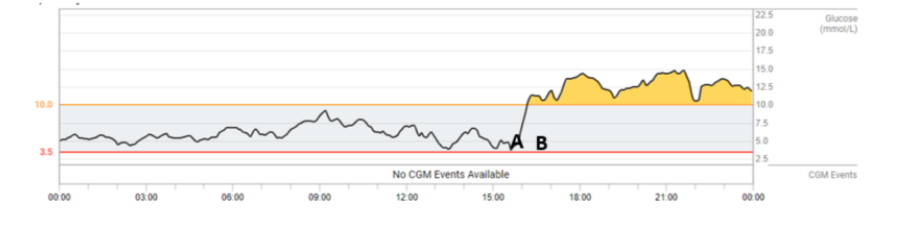
**Patient 7:** CGM trace in patient #7 undergoing Line insertion, labelled as follows: A = Surgery started, B = surgery completed.


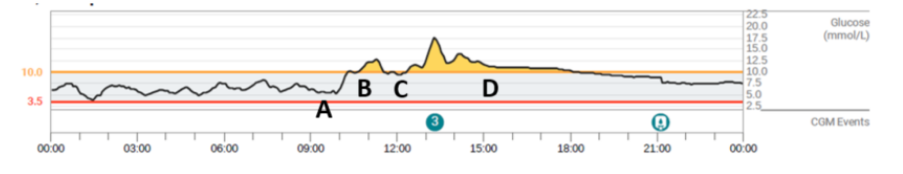
**Patient 8:** CGM trace in patient #8 undergoing Focal Lesionectomy. During surgery, the patient received continuous intravenous dextrose and did not receive medication causing hyperglycaemia A = Anaesthetic induction B = Surgery started, C= Focal lesion removed, D = Surgery completed.


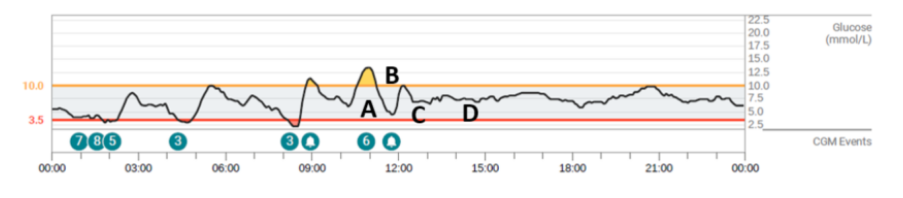
**Patient 9:** CGM trace in patient #9 undergoing Focal Lesionectomy. During surgery, the patient received continuous intravenous dextrose and did not receive medication causing hyperglycaemia. A = Anaesthetic induction B = Surgery started, C= Focal lesion removed, D = Surgery completed


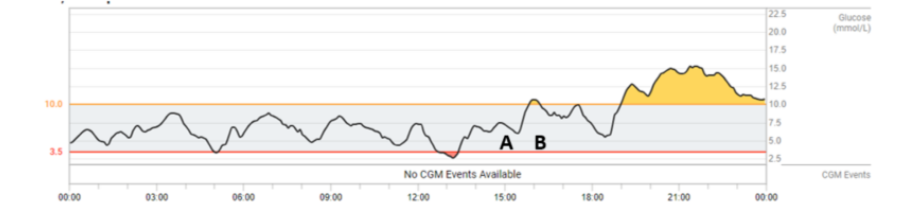
**Patient 9:** CGM trace in patient #9 undergoing Line insertion, labelled as follows: A = Surgery started, B = surgery completed


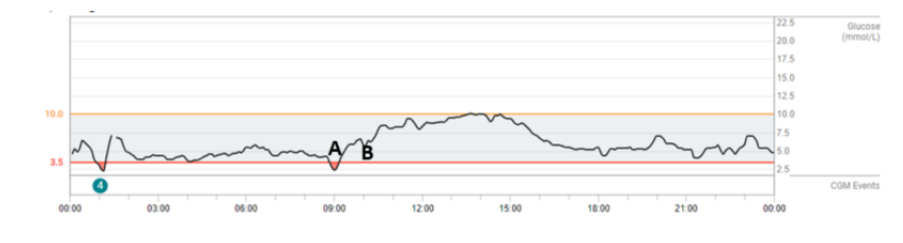

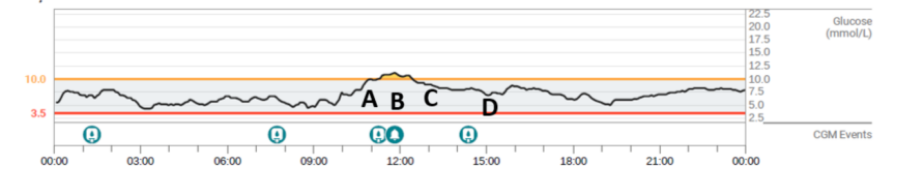
**Patient 10:** CGM trace in patient #10 undergoing Focal Lesionectomy. During surgery, the patient received continuous intravenous dextrose and did not receive medication causing hyperglycaemia. A = Anaesthetic induction B = Surgery started, C= Focal lesion removed, D = Surgery completed

**Patient 10:** CGM trace in patient #10 undergoing Line insertion, labelled as follows: A = Surgery started, B = surgery completed


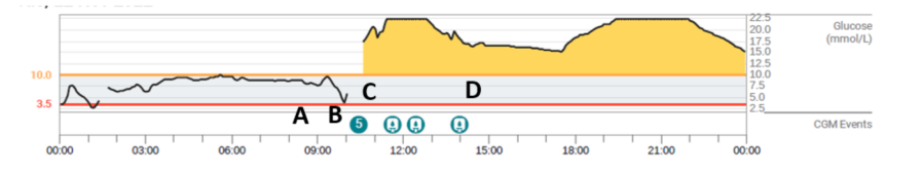
**Patient 11:** CGM trace in patient #11 undergoing a 95% subtotal Pancreatectomy. During surgery, the patient received continuous intravenous dextrose and did not receive medication causing hyperglycaemia. A= Anaesthetic induction B = Surgery started, C= 95% Subtotal Pancreatectomy, D = Surgery completed.


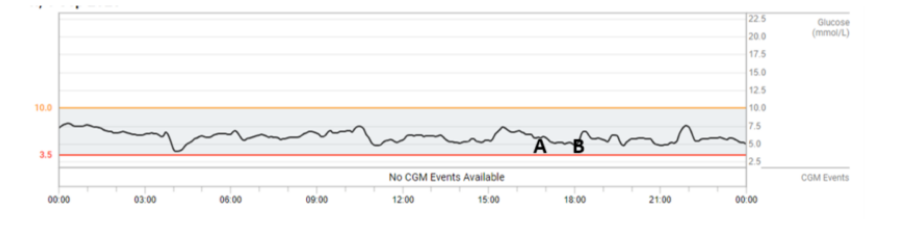
**Patient 11:** CGM trace in patient #11 undergoing Line insertion, labelled as follows: A = Surgery started, B = surgery completed


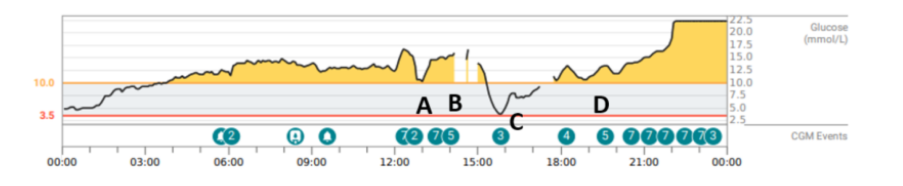
**Patient 12:** CGM trace in patient #12 undergoing Focal Lesionectomy. During surgery, the patient received continuous intravenous dextrose and did not receive medication causing hyperglycaemia. A = Anaesthetic induction B = Surgery started, C= Focal lesion removed, D = Surgery completed


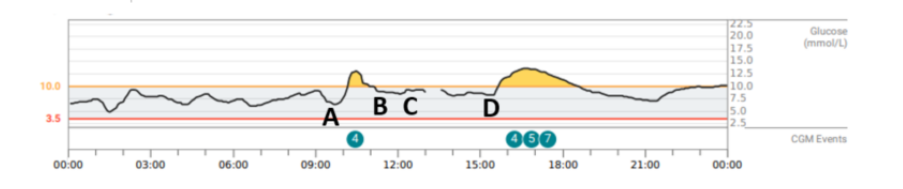
**Patient 13:** CGM trace in patient #13 undergoing Focal Lesionectomy. During surgery, the patient received continuous intravenous dextrose and did not receive medication causing hyperglycaemia. A = Anaesthetic induction B = Surgery started, C= Focal lesion removed, D = Surgery completed
